# Supplementary material for: Assessing the Risk of Occurrence of Bluetongue in Senegal
Source: Microorganisms. 2020 Nov 11;8(11):1766. doi: 10.3390/microorganisms8111766 (PMC7697801; doi:10.3390/microorganisms8111766)
Supplement: Supplementary file 1 [file microorganisms-08-01766-s001.pdf]

microorganisms

Supplementary Information:

Geographical location of sampling sites of the entomological and serological survey

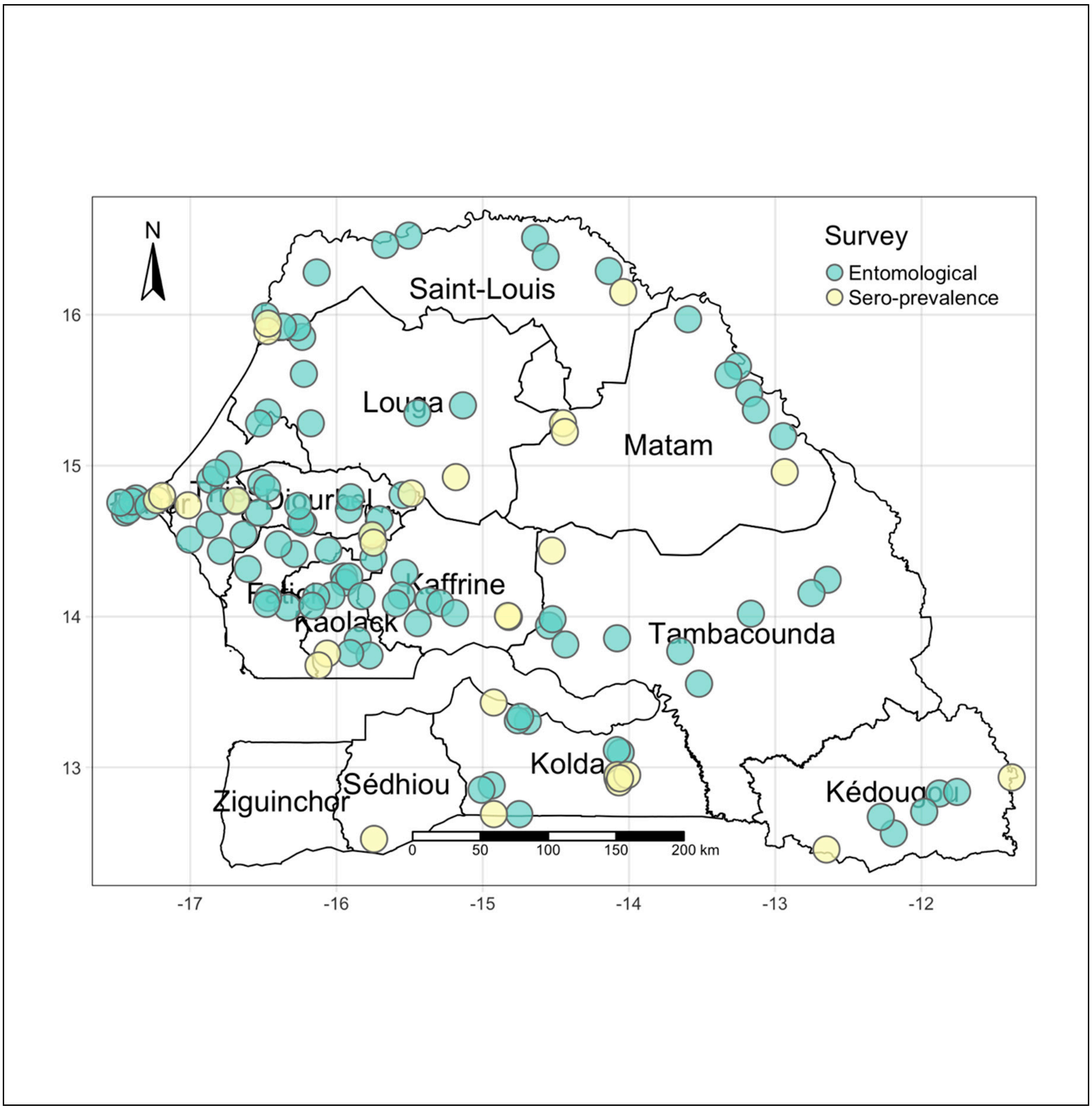

Figure S1 Sampling sites: (blue circles) Entomological capture points; and (yellow circles) for serological survey.

Results of the serological and entomological survey are shown in table where we reported the average sero-prevalence by Region, the number of veterinarian Interest Culicoides collected specimens and their distribution

Table S1. Results from the Serological and Entomological Survey by Region. The second column contains the average BT sero-prevalence in the Region, the third the number of specimens for Culicoides of Veterinarian interest, the other ones show the distribution of the 4 species .

	Serological Survey	Entomological Survey				
**Region**	Prevalence (%)	Total Specimens	C.imicola (%)	C.oxystoma (%)	C.enderleini (%)	C.miombo (%)
**Dakar**	41,9	65394	1,7	97,9	0,3	0,1
**Diourbel**	82,5	33319	40,3	10,5	10,0	39,2
**Fatick**	97,2	176236	9,8	52,4	34,5	3,3
**Kaffrine**	55	28015	9,4	6,9	19,8	63,9
**Kaolack**	50	71008	30,9	26,2	30,7	12,3
**Kédougou**	81,2	60402	57,1	4,1	34,5	4,2
**Kolda**	62,4	180473	12,7	9,6	51,2	26,5
**Louga**	95	1201	41,9	16,8	24,4	16,9
**Matam**	81,6	3361	34,4	30,3	35,4	0,0
**Saint-Louis**	86,6	13224	6,7	9,4	83,7	0,3
**Sédhiou**	39,4					
**Tambacounda**	72,2	33005	17,3	10,9	19,7	52,1
**Thiès**	41	31003	27,7	8,2	31,8	32,4

The epidemiological model that describes the dynamics of Bluetongue virus infection in small ruminants and vectors is described by the following set of related differential equations:

$$\begin{cases} \frac{dImm_h}{dt} = b_h R_h - (\eta_h + d_h) Imm_h \\ \frac{dS_h}{dt} = \eta_h Imm_h + b_h (N_h - R_h) - (\lambda_h + d_h) S_h \\ \frac{dE_h}{dt} = \lambda_h S_h - (\alpha_h + d_h) E_h \\ \frac{dI_h}{dt} = \alpha_h E_h - (r_h + d_h) I_h \\ \frac{dR_h}{dt} = (1-p) r_h I_h - (d_h) R_h \\ \frac{dD_h}{dt} = (p) r_h I_h \\ \frac{dS_v}{dt} = b_v N_v - (\lambda_v + d_v) S_v \\ \frac{dE_v}{dt} = \lambda_v S_v - (\alpha_v + d_v) E_v \\ \frac{dI_v}{dt} = \alpha_v E_v - (d_v) I_v \end{cases}$$

Table S1 List of the environmental, climatic, and animal density variables used for the analysis and their abbreviations

Category of variables	Description	Abbreviatio[illegible]
**Temperature**	Annual Mean Temperature	Bio01
	Mean Diurnal Range (Mean of monthly (max temp – min temp))	Bio02
	Isothermality (Bio02/Bio07)*100	Bio03
	Temperature seasonality (standard deviation *100)	Bio04
	Maximum Temperature of Warmest Month	Bio05
	Minimum Temperature of Coldest Month	Bio06
	Temperature Annual Range (Bio05-Bio06)	Bio07
	Mean Temperature of Wettest Quarter	Bio08
	Mean Temperature of Driest Quarter	Bio09
	Mean Temperature of Warmest Quarter	Bio10
	Mean Temperature of Coldest Quarter	Bio11
**Precipitation**	Annual Precipitation	Bio12
	Precipitation in the wettest month	Bio13
	Precipitation in the driest month	Bio14
	Precipitation seasonality (coefficient of variation)	Bio15
	Precipitation in the wettest quarter	Bio16
	Precipitation in the driest quarter	Bio17
	Precipitation in the warmest quarter	Bio18
	Precipitation in the coldest quarter	Bio19
**Altitude**	Digital elevation model	dem
**Animal density**	Cumulated density of horses, cattle, donkeys, goats and sheep	Livestock
	Cattle density	Cattle
	Goat density	Goat

	Sheep density	Sheep
	Horse density	Horse
	Donkey density	Donkey

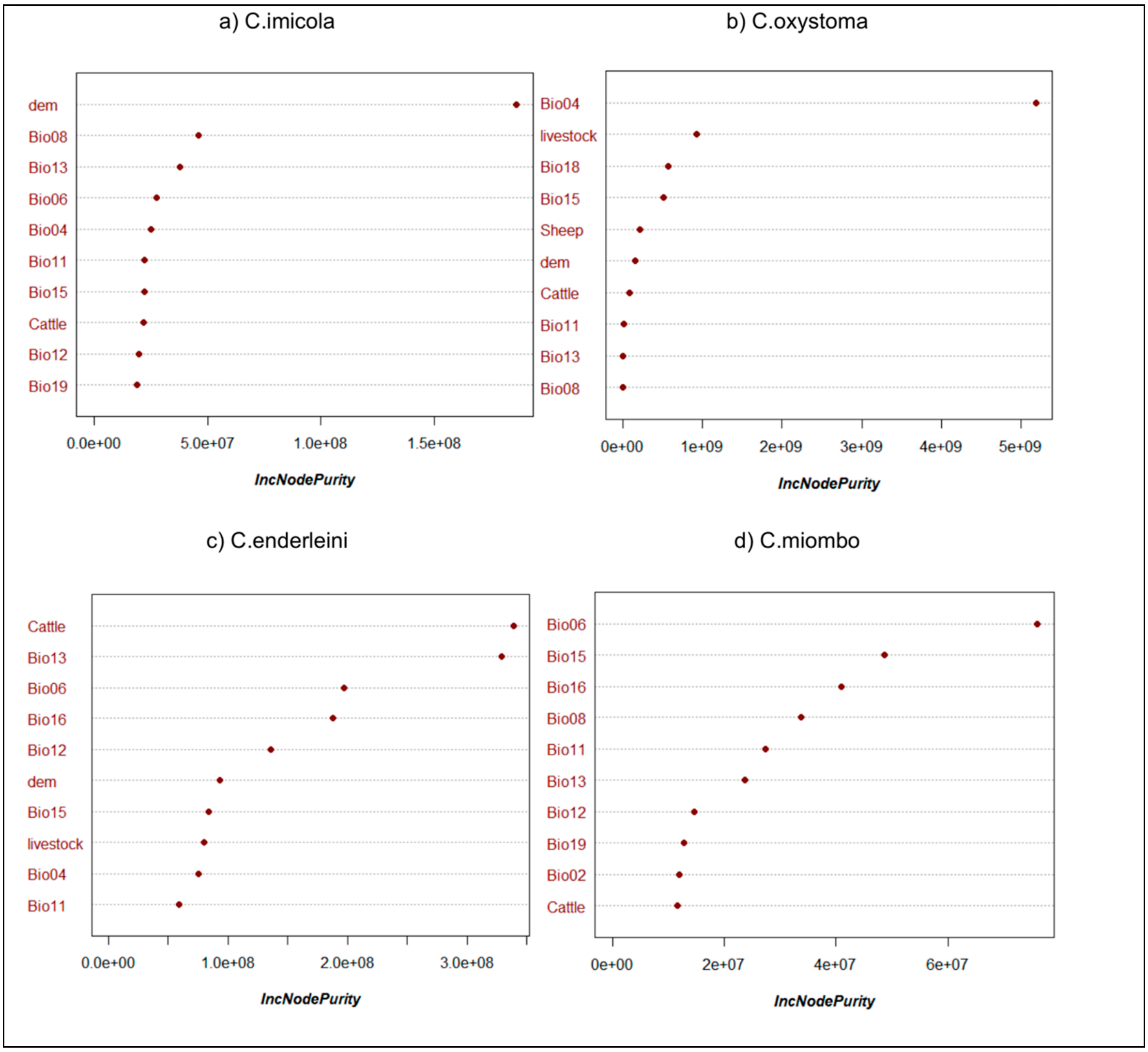

Figure S2 Importance of variables in the abundance of 4 main BTV vector species in Senegal: (a) *C. imicola*; (b) *C. oxystoma*; (c) *C. enderleini* and (d) *C. miombo*

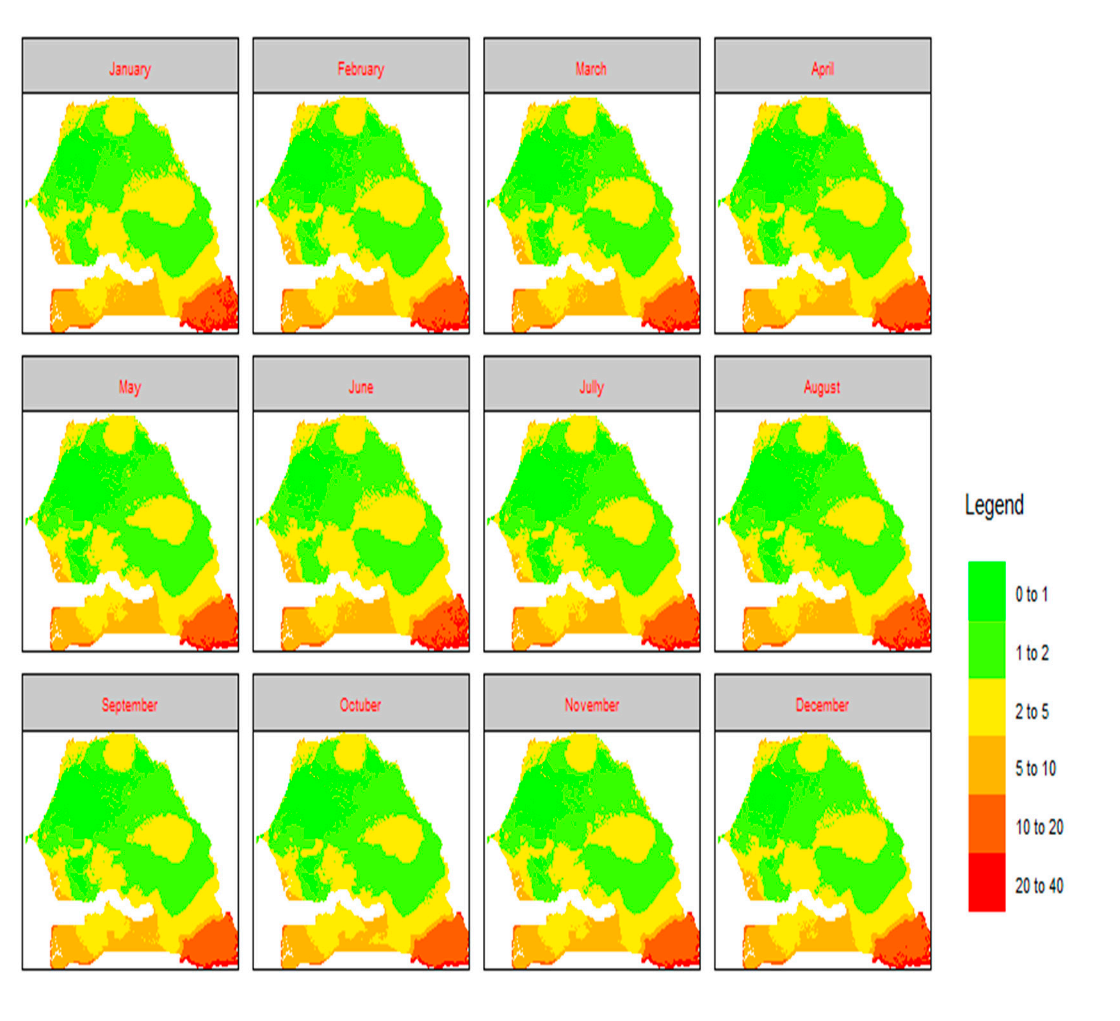

Figure S3 Monthly transmission map. Host mortality and fertility rates vary each month

Analysis of Figure S3 shows that, regardless of the month considered, Casamance (Ziguinchor, Sedhiou and Kolda) and Kédougou region have a high probability of the occurrence of a major epizootic disease as the value of R_0 is high in all these areas, regard less of the month of the year. We also note that the trends in the risk map based on the average monthly daily birth and death rates of small ruminants differ slightly. Indeed, all the other parameters remain the same from one month to another, and the differences between the risk maps reflect the differences in the average daily and monthly birth and death rates of small ruminants for the different months.
